# An integrated model for evaluation of big data challenges and analytical methods in recommender systems

**DOI:** 10.1186/s40537-022-00560-z

**Published:** 2022-01-31

**Authors:** Adeleh Asemi, Asefeh Asemi, Andrea Ko, Ali Alibeigi

**Affiliations:** 1 Department of Software Engineering, Faculty of Computer Science and Information Technology, Universiti Malaya, 50603 Kuala Lumpur, Malaysia; 2 Doctoral School of Economics, Business, & Informatics, Corvinus University of Budapest, Fovam ter 8., 1093 Budapest, Hungary; 3 Corvinus University of Budapest, Fovam ter 8., 1093 Budapest, Hungary; 4 Faculty of Law, Universiti Malaya, 50603 Kuala Lumpur, Malaysia

**Keywords:** Recommender system properties, Big Data properties, Dig Data challenges, Analytical methods, Fuzzy multi-criteria decision making, Fuzzy AHP, Fuzzy inference system, Privacy

## Abstract

The study aimed to present an integrated model for evaluation of big data (BD) challenges and analytical methods in recommender systems (RSs). The proposed model used fuzzy multi-criteria decision making (MCDM) which is a human judgment-based method for weighting of RSs’ properties. Human judgment is associated with uncertainty and gray information. We used fuzzy techniques to integrate, summarize, and calculate quality value judgment distances. Then, two fuzzy inference systems (FIS) are implemented for scoring BD challenges and data analytical methods in different RSs. In experimental testing of the proposed model, A correlation coefficient (CC) analysis is conducted to test the relationship between a BD challenge evaluation for a collaborative filtering-based RS and the results of fuzzy inference systems. The result shows the ability of the proposed model to evaluate the BD properties in RSs. Future studies may improve FIS by providing rules for evaluating BD tools.

## Introduction

Today, the COVID-19 Pandemic has led to a significant expansion in using RSs. Therefore, evaluating and comparing BD challenges and analytical methods based on the individual needs of RSs has received more attention from researchers. RSs use the data as the main input to create the recommendations. Data is created constantly, and at an increasing rate for RSs. By using RSs, a lot of new data is generated through different devices and on different platforms. “In the past few years, we have seen a lot of progress in the problem of RSs and in the problem of the lack of accurate data. However, the problem of lack of data is still very challenging [7].” The sources of new data are remarkably diverse, and all the created data are valuable and must be considered for creating recommendations. In this process, devices and sensors automatically generate data that must be stored and processed in real-time. It is certainly difficult to keep up with this huge influx of data, but what is significantly more challenging is when enormous amounts of data do not confirm traditional concepts of data structure. In this situation, it will be difficult to identify meaningful patterns and extract useful information. These challenges provide an opportunity to design new RSs that change industries, science, and everyday life. In this regard, various industries have been developed in the direction of RSs for BD through the collection and efficient use of data. Some examples are:

- Credit card companies for e-commerce track every purchase their customers make and can detect fraudulent purchases with high accuracy using rules derived from the processing of billions of transactions.
- Mobile phone companies analyze subscriber contact patterns for daily interaction with RS. They can determine if a caller’s repeated calls are found on a competing network.
- In social-based RSs that receive services from companies like LinkedIn and Facebook, data is their main product. The valuation of these RSs is strongly derived from the data they collect and host, which has increasing intrinsic value as the data grows.

The main problem of the present study is related to the challenges of BDs, included I. Huge amount of data that instead of thousands or millions of rows, BD can be billions of rows and millions of columns, II. The complexity of data types and structures is another important challenge. BD reflects a variety of new data sources, formats, and structures, including the digital footprint left on the Web and other digital repositories for later analysis. III. The third challenge is the speed with which new data can be created and grown. BD can describe high-speed data and analyze and consume data in real-time. RSs play an effective role in meeting these challenges. Although the volume of BD attracts the most attention, the variety and velocity of the data provide a more appropriate definition of BD. Sometimes BD has 3 Vs: volume, variety, and velocity. Due to its volume or structure, BD cannot be effectively analyzed using traditional RS alone. BD needs new tools and technologies to store, manage, and implement accurate recommendations. These new tools and technologies make it possible to create, manipulate, and manage the BD and storage data in related environments. Therefore, the challenges of BD in RS are not limited to "data", but we must consider the challenges of data processing and data management too. RSs have different types and properties, so the severity of BD challenges and the appropriateness of the BD analytical method varies. This study aimed to provide an integrated model for ranking challenges and analytical methods for RS. There are three main research questions:

- Q1. What is the weight of RSs properties in ranking BD challenges and analytical methods?
- Q2. How can we automatically rank BD challenges in different RSs?
- Q3. How can we automatically determine the best analytical method for dealing with BD in each different RSs?

To answer the first question, we applied the Fuzzy AHP method to weigh the importance of RSs’ properties in the ranking of BD challenges and analytical methods. Then for Q2 and Q3, we implemented two fuzzy inference systems to rank the challenges in RSs and evaluate the analytical method, respectively. The first FIS receives the type and properties of RSs and returns the ranking of all BD challenges for that specific RS. The second FIS similarly receives the properties and type of RSs and returns the rank of the analytical method. The proposed FISs help researchers and data scientists to find the most proper BD techniques to address the BD challenges in RSs.

## Related works

The present study on BD’S RS is divided into three sections:

(I) Propose an analytical method for a specific type of Zhou et al. [31] proposed an especially distributed federal contextual framework for online learning. This framework is supported by BD technology and is a social RS with privacy. Researchers also apply text and data mining to support BD in RSs [4, 5]. Chen, et al. [11] implemented a scientific RS using the Scala programming language and algorithms provided by Spark MLib to support and analyze large scientific data. Dwivedi and Roshni [14] implemented RS for BD in e-learning.
(II) Use of problem-based RS in BD. Deebak and Al-Turjman [13] proposed a framework for a trust-aware RS that supported BD cloud network reliability. They improved the structure of traditional recommending systems in terms of providing reliable data collection from users. Habibzadeh, et al. [18] proposed an integrated model to meet the challenges of 3Vs in smart cities. This model uses RS machine intelligence and data analysis together to meet BD challenges.
(III) Review of BD's RSs: There are studies about the latest version of review studies in RS considering the challenges of BD [12, 15, 23].

In this study, we proposed a model for adopting the best technique to support BD in different RSs. Therefore, this study emphasizes the automation of the third group of BD’s RS studies. In this regard, we have reviewed BD challenges in three groups of challenging data, process challenges, and management challenges.

Data Challenges are a group of challenges related to the properties of the data itself. Dif-ferent researchers have different understandings of data properties. As some researchers believe, data has three challenges (3Vs) include volume, velocity, and variety [19]. Kaur et al. [21] Considered four challenges (4Vs) for data, including volume, velocity, variety, and variability. Kaur and Sood [20] reported six challenges (6Vs) for data such as volume, velocity, variety, veracity, variability, and value. Sinaeepourfard et al. [29] by analyzing the various articles, identified seven challenges (7Vs) for data.

Process challenges are a group of challenges encountered when processing and analyzing data, ranging from data collection to interpretation and presentation of results. Since large datasets are usually unrelated or unstructured, processing such unstructured or semi-structured datasets on a scale pose a significant challenge [1, 24]. There are several data processing challenges grouped into five stages: data collection and storage, data mining and clearing, data integration and aggregation, data analysis and modeling, and data interpretation [30].

Management challenges include a group of challenges that are encountered when accessing, managing, and administering data in BD [28]. Data warehouses contain large amounts of data including both personal and sensitive data. They store data such as financial transactions, medical procedures, insurance claims, diagnostic codes, personal data, and so on. Organizations and companies must ensure that they have a strong security infrastructure that en-ables employees and employees of each department to view only relevant data for their department. In addition, there should be some standard privacy rules that may govern the use of such personal information, and strict adherence to these privacy policies should apply to the data warehouse. Organizations attach different importance to different types of data challenges. For example, in the health industry, the most important challenge for BD management is privacy [31]. There are several challenges related to data management, which are categorized into seven areas such as privacy, security, data and information sharing, cost/operational costs, data governance, and data ownership [30].

BD analytical methods can be seen as a sub-process in the overall process of extracting insights from BD. Despite advertisements for various BD Analytics (BDA) methods, the use of analytics is still an intensive task. Assunção, et al. [8] argue that current solu-tions for BD analysis are often based on proprietary tools or software systems built for general purposes. As a result, RSs need to be customized to meet their needs, just like BDA solutions, which can be done based on RS features. In this study, an FIS is imple-mented and in this fuzzy system, considering RS properties, BD challenges, and its ana-lytical methods are ranked.

Current studies highlight several analytical processes and methods such as text analysis, audio analysis, video analysis, social media analysis, and predictive data analysis [17]. Given certain problems such as uncertainty in analytical methods by Hariri, et al. (2019), Some researchers reported descriptive data analysis, inquisitive data analysis, prescriptive data analysis, and preemptive data analysis (Willetts, Atkins, et al. 2022). In these different methods of BD analysis, there are several off-the-shelf software tools such as Hadoop, MapRecuce, Dyrad, which are built using existing software and their expansion, and finally, new solutions to deal with BD analysis, for example, have presented an integrated model for BD [13, 18]. Sivarajah et al. [30] identified and classified analysis methods into three groups including descriptive analysis, predictive analysis, and prescriptive analysis. However, nothing has been specifically considered for inquisitive and preemptive analysis.

## Methodology

**Fig. 1:**
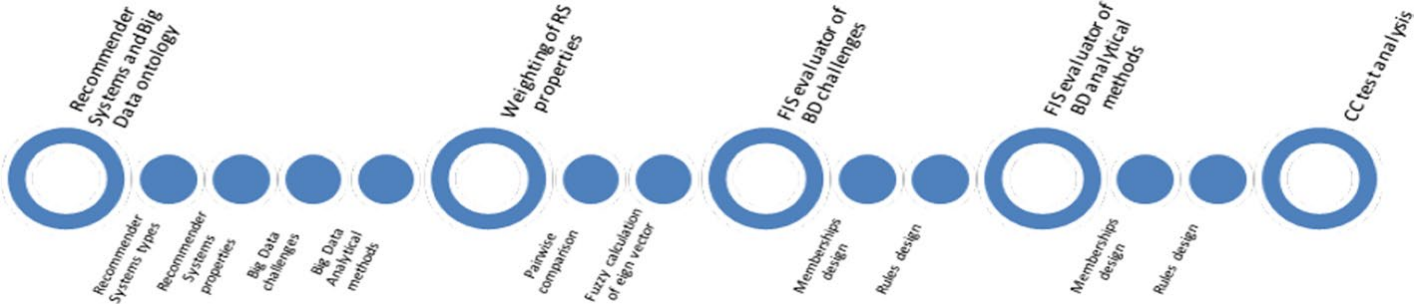
Proposed model

In this study, we proposed an integrated model (Fig. 1) based on two decision-making techniques as fuzzy MCDM and FIS. This model first provided the required attributes and properties in BD and RS which are important in BD RSs' decision-making. Therefore, in the first step, the RS and BD ontology is considered. Then fuzzy Analytical Hierarchy Process (AHP) is applied for the weighting of evaluation criteria (RSs properties). In fuzzy AHP, provided a pairwise comparison matrix using the eigenvector calculation to provide the weights of properties. This ranking is used to design rules in FISs. Two FISs are designed for ranking BD challenges and BDA methods based on the individual needs of RSs. The researchers and data scientists can use FISs to get recommendations for the best BD technique in RSs.

### Ontology of RS

The information system is any kind of system where a lot of information is stored, and this information system is equipped with RSs. It uses RS techniques and methods to meet the information needs of the users or end-users. RSs are a subset of information systems with the ability to interact with users as well as product recommendations for users. RSs interact with other information systems and users to receive information and send the output as recommendations. RSs are software tools and techniques that provide suggestions for items that are of interest to a particular user [9, 25–27]. There are several basic rules related to the design of the ontologies, but all include the determination of (1) ontology development methodology, (2) ontology language and (3) ontology development environment (tool). Ontology development is usually a repetitive, iterative process because the users must reach a consensus about it. The literature describes several types of methodology that aim expressly in the planning of ontology. Our development method followed the Menthology approach [16]. The stages of this methodology are specification, knowledge acquisition, conceptualization, integration, implementation, and evaluation that is an emphasized stage of Methontology. In this study, we have focused on the conceptualization of the RSs and the relation between the RSs' properties. Here, the ontology is implemented in Protégé 5.5, the figures, visualization were prepared in OntoGraf and OWLViz. This section presents the main elements of the RSs' properties ontology, their relations, and descriptions. General RSs' ontology includes the following objects:

- Axiom count 287
- Logical axioms count 98
- Declaration axioms count 103
- Class counts 87
- Object property count 9
- Data property count 3
- Individual count 4
- Annotation Property count 2
- Sub Object Property Of 2

**Fig. 2:**
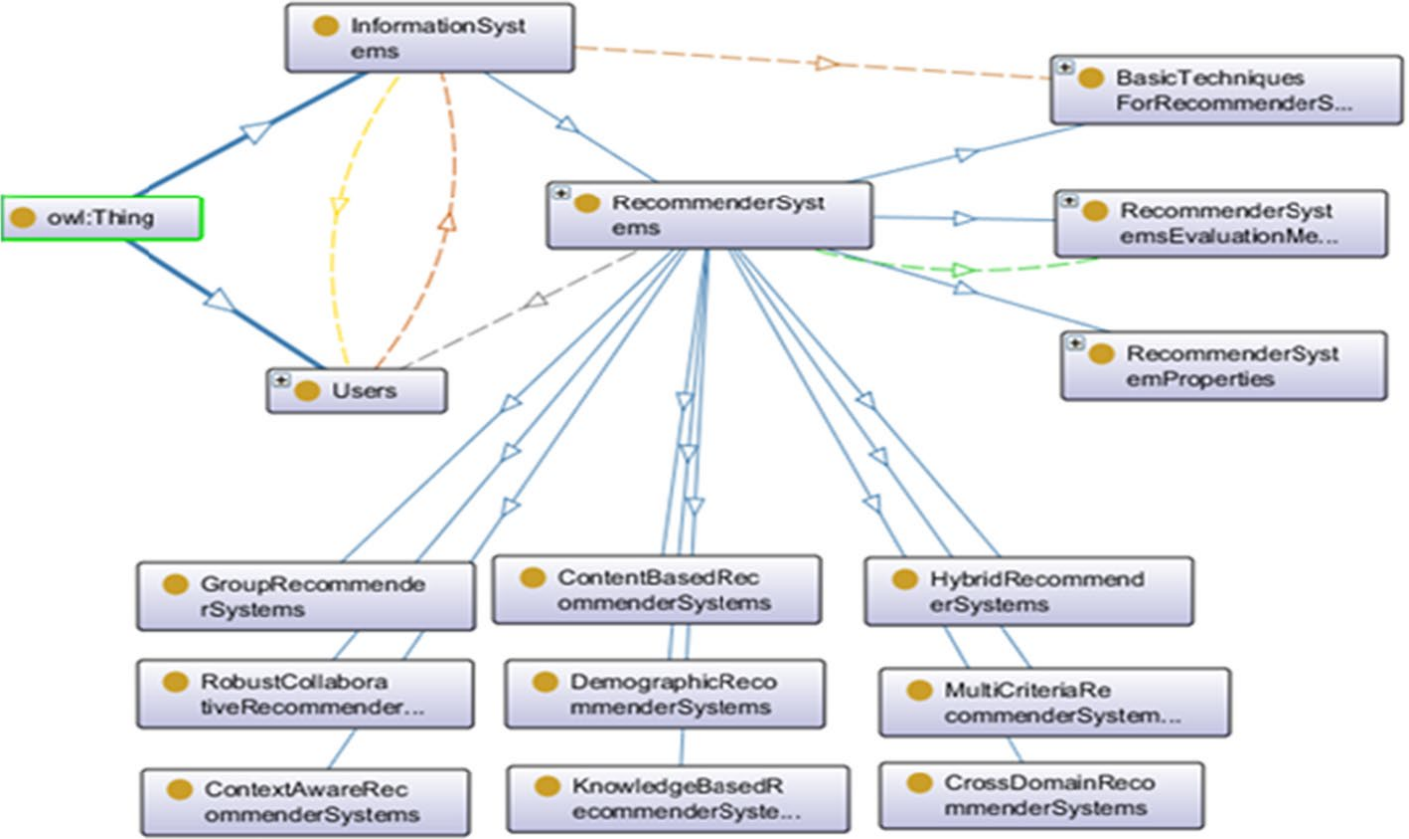
General OntoGraph of RSs

**Fig. 3:**
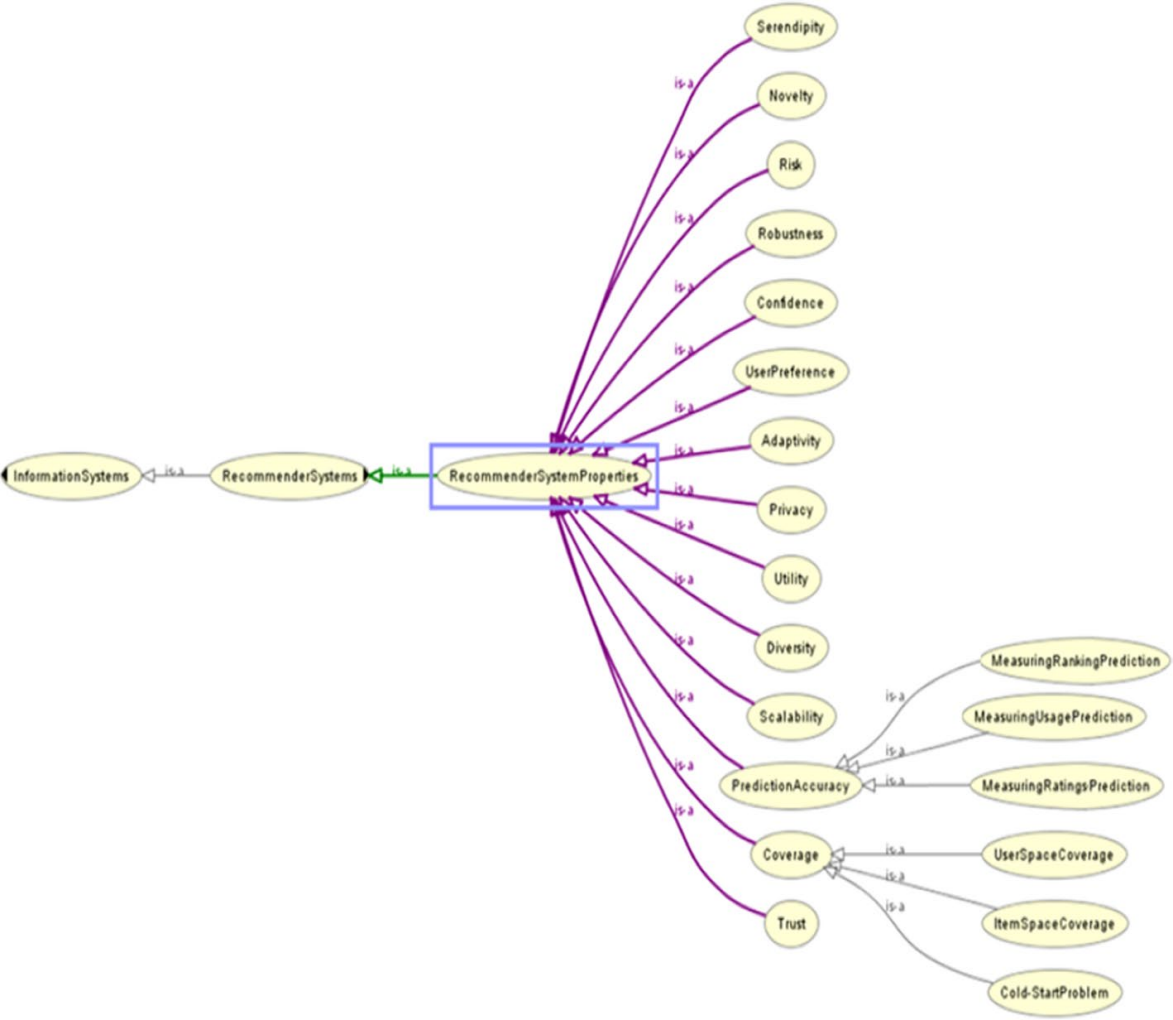
General ontology of RSs' Properties

Figure 2 shows the general ontology of RSs. RSs’ properties are considered when we decide to select RS’s type. Evaluation of RSs is required to be performed at various stages and time intervals of the system cycle. This evaluation is done for different purposes. Figure 3 shows an OWL visualization of the RSs’ Properties. All components are described as follows.

Regarding user preferences, the suggestions that RSs provide to users should be evaluated. These suggestions should be considered to what extent they match the preferences of system users and to what extent it has helped users to make decisions. It should also be examined to what extent RSs make it easier and faster for users to find preferred items. In implementing RSs, useful items for users should be identified based on their current needs and potential needs. It should be checked how much an item is worth recommending to the user. To do this, RSs must be able to predict the usefulness of the proposed items. In this regard, the minimum usage of the items is compared with each other and then it is decided which item or items are more in line with the preferences of the user or users. In knowledge-based RSs, the features of the recommended items are considered for the potential and actual users. These features are based on the specific domain knowledge of the items that match the users' preferences as much as possible. The recommendations show how useful they are for users.

An RS is designed for the users' utility. The user's behavior has a key role in the evaluation of the RS. We cannot evaluate an RS without considering users' utility as separate classes. For this reason, we considered utility as an important property for the RSs.

Usually, a user has sought to find an answer to her/his needs. When the user's needs are met properly and quickly, the user finds a confident feeling than RS. Hence confidence is another important property that must be considered for the RSs.

Knowledge-based RSs operate case-based. The degree of compliance of the recommendations is estimated and displayed than the needs or preferences of the user. Serendepity, in one method, the adaptability of a recommendation can be checked by analyzing the amount of information required before recommending an item. Another way to check the adaptability is to check the compatibility of the recommendations with the user's personalized preferences in their profile.

The assurance of recommendation of the RSs can be considered as the system's trust in the recommendations or predictions it makes. As a rule, the higher the adaptability of the system, the greater the level of trust in the RSs. These two properties are related to each other.

Coverage addresses the range of issues that the system recommends to the user. In this regard, it includes userspace. Coverage can also include all the items that RS decides or predicts. Coverage can vary depending on the goals of the system. Usually, the term coverage refers to the proportions that RS can recommend. It can refer to several distinct features of the system. One of the sub-properties of coverage is the "cold start". Users' needs and preferences change over time. Also, the set of items that the system recommends changes over time. Therefore, the coverage property usually has a cold start, and it is necessary to restart the system from the beginning. At the beginning of each cold start in RS, the system must upload and re-implement the updated data to provide appropriate recommendations. Coverage can also be related to the ratio of users or their interactions to items that the RS recommends.

Variety is another RSs' property that can apply to items, users, and recommendations to users. RSs always need to pay attention to the diversity of services they provide. Also, users may have a variety based on the goals of the RS.

**Fig. 4:**
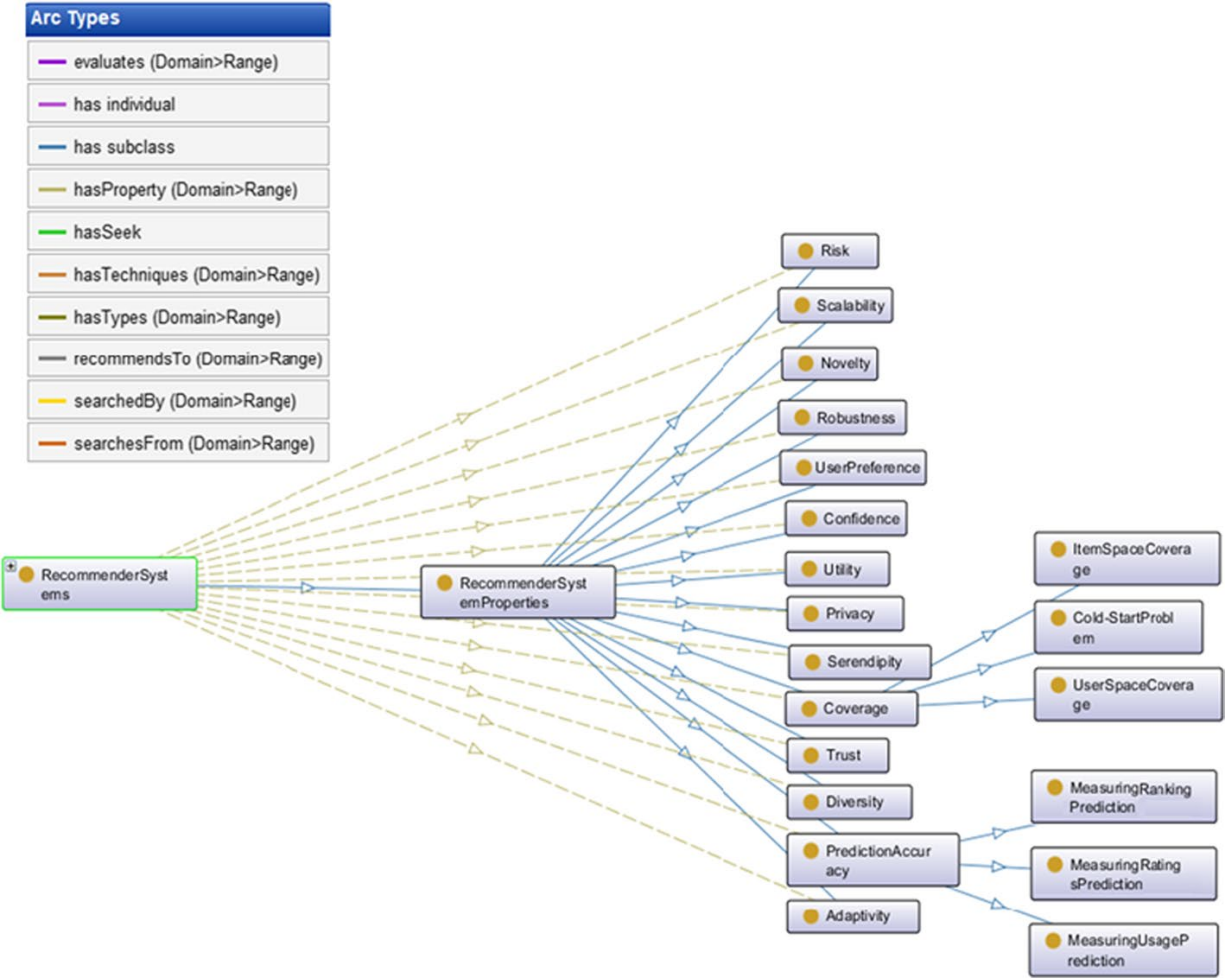
General ontology of RSs' Properties (OntoGraf)

As you can see in Fig. 4, predictive accuracy is another property of the RSs. The accuracy of the prediction should be such that it also considers the potential needs of the users. Using users' feedback on recommendations can help increase prediction accuracy. In this case, the prediction will be based on user preferences. Predictive accuracy can include ranking, rating, and usage of the recommendations provided by the RS. As the accuracy of ranking, rating, and usage increases, the accuracy of the prediction will increase.

With the advancement of technology and the increase of data, RSs need to always be innovative to provide customer-friendly services. In this regard, high quality and novelty is essential for RSs. In addition to being a novelty, these systems require algorithms that are robust enough to reduce system error in making recommendations. In the robustness assessment process, the performance of RS is checked to see whether the system performs well in different conditions.

Many people have the experience of getting tired of over-recommendations by RSs. Such as the recommendations that appear on the web page when using different websites and cause annoyance to the user. These recommendations may relate to the user's previous needs and now have no role other than annoying the user. To solve this problem, recommending systems must suggest serendipitous items. According to Kotkove et al. [22], offering serendipity items involves certain challenges. Designing a serendipity-based RS with a proper algorithm requires selecting appropriate objectives. However, being serendipity is another property of RSs. It can be a measure of the amazing success of successful advice.

Privacy is one of the most important properties of RSs. Maintaining and protecting information about users' preferences is particularly important. It is necessary that no third party log in to the system to receive users' information.

The risk properties concerning the recommendations that the system makes can be a key factor in attracting or separating the user from the system. In some cases, even a recommendation from the system may be associated with a potential risk. Since fake information is abundant, it is necessary to consider the strength and stability of the recommendation in the presence of fake data. It should be noted that the impact of this kind of data and information on recommendations is important.

As the data grows, the scalability of the system is considered. Also, increasing the number of recommendations is effective in scaling the system.

### Ontology of BD

**Fig. 5:**
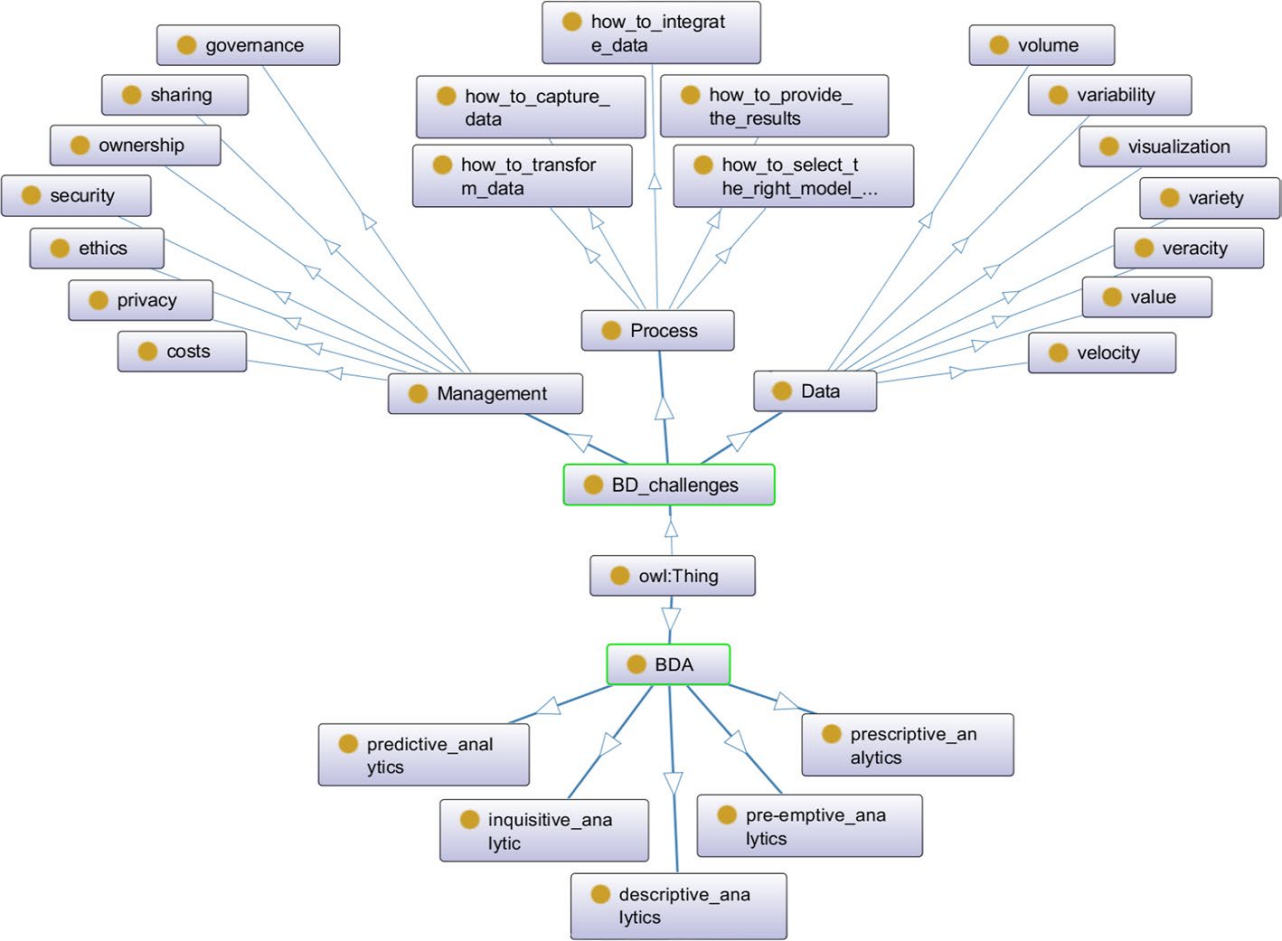
General ontology of BDs' Challenges (OntoGraf)

The challenges of BD and BD management methods can be considered as key points for designing the best technique of BD-based systems. Figure 5 shows the overall ontology of BD and the relationship between its components.

Extensive BD challenges can be categorized into three main categories based on the data life cycle [30]: Data, process, and management challenges.

Each of these challenges has its sub-challenges. Data sub-challenges relate to the properties of the data itself. Some of the important data sub-challenges include data volume, Va-riety, Veracity, Velocity, Variability, Visualization, and Value. The sub-challenges of the BD processes include a set of techniques and activities that are addressed in BD. Such as how to collect data, integrate data, convert data, data analysis model, and how to present the results are also among the process challenges in BD. The challenges of managing BD include some issues of privacy, security, governance, sharing, ownership, costs, and ethics. Process challenges in BD have led to the use of various methods for Big Data Analyt-ics (BDA). The five major categories of methods are descriptive data analysis, inquisitive analysis, predictive analysis, prescriptive analysis, and pre-emptive analysis.

The following is a brief definition of components in BD ontology related to sub-challenges of data challenge:

- Volume: In BD, the size of the data set is usually petabytes, zettabytes, and even more. The high volume of data is a big challenge in BD. On the other hand, the heterogeneity and pervasiveness of the data are controversial. Also, the dynamic nature of data generation and the use of various devices with various applications create different challenges. These challenges are typically related to data retrieval, processing, integration, and inference. Therefore, the various methods and techniques used for this purpose require new and novel approaches.
- Variety: Another BD challenge, like any other database, is the challenge of diversifying data into different formats. These formats may be structured or unstructured. BD may include a variety of data, including textual, visual, audio, multimedia content, sensor data, and other data. Studies show that usually a large amount of data is not compatible with the system and does not follow a specific format. For example, receiving user feedback may be presented in different forms and using different tools.
- Veracity: In this challenge, BD often faces problems with complex data structure, anonymous data, and inaccuracies in the data. This challenge is about data quality and how to understand data. Some data are inherently unreliable, and some are unstructured, so BD tries to develop various data analysis techniques and tools and apply appropriate behavior to types of data to maintain data veracity and increase data set accuracy.
- Velocity: This challenge is usually related to the high rate of data flow with a heterogeneous structure. BD needs to manage high data influx rates to meet this challenge. Data is usually heterogeneous but with proper management can generate new data and update the system [11]. This is especially true for those datasets that are generated through large complex networks. For example, real-time personalized RSs are commonly used to manage user data.
- Variability: In this challenge, BD is always faced with changing data. An example of a solution to this challenge is to analyze users' emotions and sentiments. Using the right algorithms is especially important in this case. These algorithms must be able to understand the context and decode the exact meaning of a word in that context (Zhang et al. 2015). Nevertheless, this issue is still particularly challenging.
- Visualization: One of the most important challenges is BD. Data visualization plays a significant role in decision-making. Data visualization is used as decision support for BD's users and managers. Reading data is one of the ways to visualize data. Visualizing data about key information and knowledge is more effective than using various visual formats such as graphics data. By visualizing the data, the user's interaction with the system can be examined and the search quality can be visualized. Visualization of data from user feedback and their emotions is a clear example in this regard.
- Value: The data value challenge is about extracting knowledge from data. This value is obtained from a large amount of different data. Extracting information and knowledge from word search in Google is a significant example that using Google Trend can be viewed statistics and analysis about the amount of presence those words in Google search.

Process challenges in BD are the challenges that BD faces when processing and analyzing data. These challenges continue from the time of data collection and even before that to the interpretation and presentation of the results. There are five important steps in the BD process as follows:

- Step 1. Data collection and storage: This challenge is related to accessing data from various sources and storing it for different purposes.
- Step 2. Cleaning and Data Mining: This challenge is about extracting and cleaning data from a large-scale unstructured data set.
- Step 3. Data aggregation and integration: This challenge relates to the collection and integration of cleaned and extracted unstructured data from BD.
- Step 4. Data Analysis and Modeling: This challenge relates to the next step after data collection, storage, extraction, purification, and integration. At this stage of the process, data analysis and modeling for BD are performed.
- Step 5. Interpretation of data: In this step, various methods and tools are used to understand the analyzed data. In this stage, the findings of data analysis and modeling results are presented to decision-makers to interpret the findings to extract sense and knowledge.

In BD, management challenges include a group of challenges that BD faces when accessing, managing, and managing data. Data warehouses contain enormous amounts of personal andsensitive data related to various transactions that must be professionally managed. The various challenges of BD management are as follows:

- Privacy: There are major privacy concerns in BD. Information privacy and data protection are the most important legal concerns and challenges for both lawmakers and the ICT industry. Privacy and data protection laws recognize the rights of individuals over their personal information and consider the individual’s consent as the main and first prerequisite for any data collection and processing. Data protection laws provide principles and standards to be observed by data users while collecting, storing, processing, disclosing, and deleting personal data. Furthermore, non-compliance with these standards and requirements will cause heavy criminal punishments. The EU General Data Protection Regulation (GDPR) is the latest and up-to-date document based on the recent technological advancements entered into force on 25 May 2018. It has provided for extraterritorial jurisdiction; in fact, it exports data protection rules outside of Europe. Data users need to consider the GDPR developments and gold standards. It is very much effective for organizations to apply new approaches to data protection like privacy by design, Data Protection Privacy Impact Assessment, data breach notification, and Data Protection Officer (DPO). This will lead to being more accountable and compliant with related laws and regulations. Finally, training programs for the management, employees, partners, and customers play a key role in line with compliance with the privacy and data protection requirements. It is much beneficial and practicable if the data users develop a self-regulatory policy in line with the requirements of the laws, data security policy, security breach policy, quarterly data protection audit, data sharing policy, data transfer policy, and importantly a standard, easily accessible and understandable privacy policy for data subjects [2]. These policies must update regularly in line with technological developments and newly developed regulations. This would increase the level of compliance and reduce the risk of responsibilities for the data users. Although huge investments have been made in this area, organizations still face challenges in managing privacy issues.
- Security: Securing your BD involves several challenges. Given the fact that most of the data generated is unstructured and cannot be processed directly by systems, organizations must use technologies that are able to integrate different data sources to protect the lives and property of individuals. In other words, these technologies must be able to retrieve data from various sources and integrate them in an integrated manner. Besides privacy, security is one of the important requirements mentioned by data protection laws. Data users must provide suitable and up-to-date security technologies and infrastructure to ensure the complete protection and safeguarding of personal information. Security includes both software and hardware equipment.
- Data governance: Always creating an appropriate governance model depends on the level of maturity of the organization for data-based decision-making. BD governance includes "procedures", "guides" and "proper management of decision making". Organizations must ensure that standard and comprehensive data is collected and that protection principles are applied to the portion of the data that they need.
- Data sharing: In BD management, sharing issues must be balanced and controlled to maximize their impact. Maintaining and sharing large volumes of data is a complex task, because, in the case of international cooperation, the security and privacy of the data may be disputed for the parties. Therefore, there is a need for an economic model that can solve economic challenges. This model should also be able to help streamline data-driven research processes.
- Costs: One of the purposes of BD technology is to minimize costs to evaluate the value of data. This is while the continuous increase of data in all different forms has led to an increase in demand for BD processing in complex data centers.
- Data Ownership: Data ownership in BD is a complex issue. Although it is difficult to store, maintain, and manage data, sharing data is extremely easy and real-time. Data ownership poses a vital and ongoing challenge, especially in the field of social media, such as who owns the data in these spaces. Both (users and social media providers) are thought to own the data. This is done by owning the item or controlling and ensuring its accuracy.
- Legal and Ethical Issues: Most legal and ethical issues in BD revolve around data. In addition to ownership and privacy, there are issues with data usage. To use the data, it is necessary to obtain licenses from government agencies.

The major categories of BDA methods are explained as the following:

- In descriptive data analysis, the current state of a BD is examined to provide developments, patterns, and exceptions in the form of standard reports, interim reports, and alerts. Descriptive analysis is the simplest form of the BDA method and involves summarizing and describing knowledge patterns using simple statistical methods such as mean, median, mode, standard deviation, variance, and frequency measurement of specific events in BD streams.
- In prescriptive data analysis, optimization and random testing are used to evaluate-ate how BD is doing. Using this method, BD tries to improve its service level and at the same time reduce costs. This type of analysis is performed to determine the cause-and-effect relationship between analytical results and BD process optimization policies. Therefore, for descriptive analysis, BD optimizes its activity process models based on the feedback provided by analytical forecasting models.
- Factor analysis is commonly used in inquisitive data analysis. This method is mostly used to approve/reject commercial offers.
- In predictive data analysis, various methods are used to predict and statistical modeling to determine future probabilities. This analysis deals with forecasting and statistical modeling to determine future probabilities based on supervised, unsupervised, and semi-supervised learning models.
- In preemptive data analysis, the purpose is to take preventive measures against events that may adversely affect BD performance. This method may be used to identify potential hazards.

### The weighting of RSs properties (Q 1)

The RSs have different properties based on their individual needs [10]. The RS properties have different importance in the determination of the severity of BD challenges. When a company needs a RS with scalability, it shows that supporting BD challenges is very important for this RS. We use fuzzy MCDM for the determination of RS properties’ importance degree. Among Fuzzy MCDM techniques, we choose Fuzzy AHP as an accurate and simple MCDM method [6]. We have 14 dimensions in our MCDM problem because of 14 RSs properties. Fuzzy theory is applied to deal with the simulation of human judgment in pairwise comparisons.

#### Pairwise comparating RSs properties

The Pairwise comparison matrix is constructed to compare the RS properties for effectiveness in the determination of challenges’ degrees. The intensity of RS properties in BD challenges is determined based on our judgments through linguistic variables (Table 1).

**Table 1:** The linguistic variable scales and related fuzzy numbers

Linguistic variables	Related fuzzy number
Very Strong (VS)	(7, 9, 10)
Fairly Strong (FS)	(5, 7, 9)
Strong (S)	(1, 3, 5)
Equal (E)	(1, 1, 1)
Weak (W)	(1, 1/3, 1/5)
Fairly Weak (FW)	(1/5, 1/7, 1/9)
Very Weak (VW)	(1/7, 1/9, 1/10)

The relative intensity of one RS property over another property for ranking in BD challenges is expressed using pairwise comparisons. These comparisons construct one pairwise comparison matrix. Let $C= \left[ {{\text{C}}_{\text{i}} } \right]_{{\text{n}}} \quad {\text{i}} = 1,\, 2, \ldots ,{\text{n}}$ be the set of RS properties. The result of the pairwise comparison is summarized in an evaluation matrix as follows (Eq. 1):

1 $$CW = \left[ {\begin{array}{*{20}l} {\text{CW}}_{11} \hfill & \cdots \hfill & {\text{CW}}_{1{\text{n}}} \hfill \\ \vdots \hfill & \ddots \hfill & \vdots \hfill \\ {\text{CW}}_{\text{n}1} \hfill & \cdots \hfill & {\text{CW}}_{\text{nn}} \hfill \\ \end{array} } \right]$$

where $CW={{[\mathrm{cw}}_{\mathrm{ij}}]}_{\mathrm{n}\times \mathrm{n}}\mathrm{and}$ ${\mathrm{cw}}_{\mathrm{ij}}$ shows the intensity of the property ${\mathrm{C}}_{\mathrm{i}}$ over property ${\mathrm{C}}_{\mathrm{j}}$ through defuzzificating fuzzy values.

#### Obtaining eigenvector

We produce the eigenvector from the pairwise comparison matrix to determine the ranking of RS properties. We apply squaring, summarization, and normalization operations on pairwise comparison, matrix to obtain the eigenvector (Eqs. 2 and 3):

1. Squaring pairwise comparison matrix and construct S as $S={{[\mathrm{s}}_{\mathrm{ij}}]}_{\mathrm{n}\times \mathrm{n}}$.
2. Summarization row elements of matrix S and construct $\overrightarrow{CS}={{[\mathrm{cs}}_{\mathrm{i}}]}_{\mathrm{n}}$ where:
  2 $${\mathrm{cs}}_{\mathrm{i}}=\sum_{\mathrm{j}=1}^{\mathrm{n}}{\mathrm{S}}_{\mathrm{ij}}$$
3. Normalization vector $\overrightarrow{CS}$ to reach eigenvector $\overrightarrow{CN}={{[\mathrm{cn}}_{\mathrm{i}}]}_{\mathrm{n}}$ where:
  3 $${cn}_{k}= \frac{{\mathrm{CS}}_{\mathrm{k}}}{\sum_{\mathrm{i}=1}^{\mathrm{n}}{\mathrm{CS}}_{\mathrm{i}}}$$
4. Repeat steps 1–3 and compares the unique vector in each iteration with the previous step to make the difference between the special vectors much smaller. The last special vector is the priority vector.

Previous mathematical studies have shown that special vector solutions are the best approach to obtain priority rankings from the pairwise comparison matrix. Therefore, values of RS properties will obtain from the eigenvector $\overrightarrow{CN}$. The appropriate vector is the priority of RS properties in evaluation BD challenges. The vector obtained for comparison matrix includes the rank of the RS priorities. We use online AHP calculation to obtain the final weights of properties.

### Fuzzy inference system for the ranking BD challenges (Q 2)

The RS properties are the needs of different RSs. All RSs should not necessarily support all the determined properties. For example, a professional RS that is designed for some specific experts does not have the property of adaptivity. In this part, we present the process of implementing FIS for data challenges as a sample. We have the same process of implementing FIS for process challenges and management challenges.

First, we determine the severity of data challenges that are caused by all RS properties. Table 2, with a five-level scale, shows how the data challenges in each RS property are important. For instance, when we have simple RSs with a lower level of complexity are adoptive. Moreover, seeing the graphics instead of numbers and receiving the maximum benefit from RSs are very interesting for users. Therefore, volume, visualization, and value are the most challenging for adaptivity. Another example is information sharing and social media-based RSs that contain privacy property [3], these RSs have a high level of data challenges in terms of volume, variety, velocity, and variability.

**Table 2:** RS properties and related challenges

RS properties	Volume	Variety	Velocity	Veracity	Variability	Visualization	Value
Adaptivity	**					****	****
Scalability	*****	***	***	***	**		
Robustness	***	*	*****	*****	***		
User Preference	***	*****		****		***	
Diversity	*****	*****	*****	**			
Confidence	*		***	*****	****		
Coverage	****	*****	***				
Trust	***	**		*****	*****		
Serendipity	***	***	***	***	***	***	***
Utility	****	****				****	
Novelty	****	*****		****	****		
Prediction Accuracy	***			*****	****		*****
Privacy	*****	*****	*****	**	****		
Risk	*****	*****	*****	*****	*****		

**Fig. 6:**
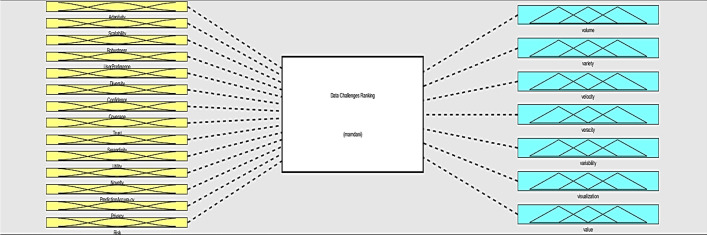
A general view of FIS for data challenges ranking

We used the RS properties as input variables of FIS and the data challenges are the output variables (Fig. 6). The FIS system receives the properties of RSs and analyzes the properties based on designed rules. Then determine the severity of all data challenges for that RS. This system is dynamic and could be used for ranking of challenges in any RS.

In this study, FIS was designed for the evaluation of challenges using MATLAB with a fuzzy logic toolbox. We implemented a Mamdani-based FIS. This system was designed to measure the influence of the RS properties on the BD challenges. In this method, a fuzzy control strategy is used to plot the given inputs through rules, and produce an output based on these rules. The input variables are fourteen RS properties, and the output variables are the BD challenges.

**Fig. 7:**
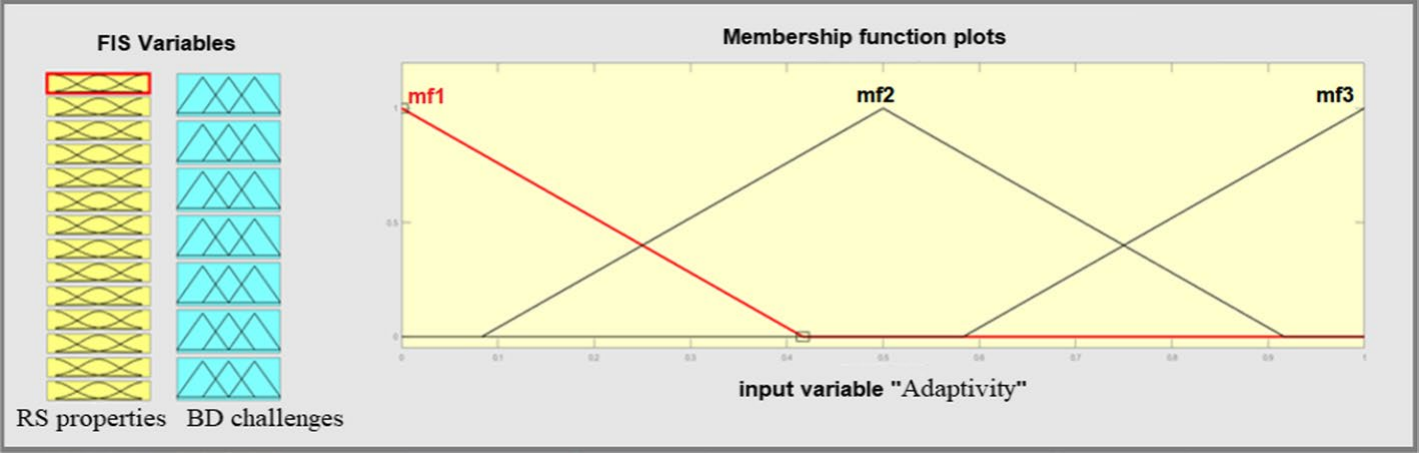
Input variables MFs

The designed system is based on fuzzy Membership Functions (MFs) and if–then rules. The MFs and generated rules help to fuzzy and eliminate fuzzy variables, which is called fuzzification. In fuzzification, perform the process of converting a fuzzy output to a clear output in FIS. The input for the FIS is a fuzzy set, and the output is a single number. An MF is a curve with membership rates between 0 and 1. The MF represents a fuzzy set and is usually denoted by μA. In the fuzzy set, for an element x of X, the value of μA is called the membership degree x. Membership degree, μA (x) determines a degree of membership of the element x in the fuzzy set. A value of 0 shows that x is not a member of the fuzzy set. A value of 1 shows that x is a full member of the fuzzy set. Specifies values between 0 and 1 indicate the fuzzy members. Fuzzy logic has eleven internal MFs, and these functions are made up of several essential functions, including linear fragment functions, Gaussian distribution function, sigmoid curves, and quadratic & cube polynomial curves. We determine the MFs for RS properties inputs and usability metrics output according to the suitability of MF in representing fuzzy variables. Figure 7 shows the designed MFs of inputs which are triangular MFs. We assign three triangular MFs for all input and output variables because triangular MF is the simplest MF. If we apply another type of MF the system complexity will be very high. Moreover, the triangular MFs can support the human opinions for determination of degree of BD challenges.

The output variables have the same MFs. A three fuzzy scale of importance representing three triangular curves was applied to determine the importance degree for each challenge.

**Fig. 8:**
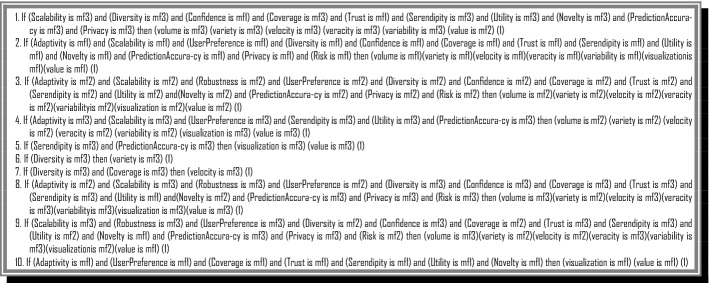
A Part of if–then Rules in the FIS

Finally, we design if–then rules in the relation between RS properties, and their effect to predict the ranking and the severity degree of BD challenges using fuzzy inferencing (Fig. 8).

### Fuzzy inference system for the ranking of BDA methods (Q3)

The requirements and properties of RSs are important in determination of suitable BD Analytical (BDA) method. Even a very complex and new technology or method could not be proper for a specific RS. Therefore, the best BDA method is the most suitable method. As we explained in BD ontology, the BDA methods are classified into five groups of predictive analysis, inquisitive analysis, descriptive analysis, pre-emptive analysis, and prescriptive analysis. In each group we there are a lot of analytical techniques that could be adopted based on data scientists’ data analytical.

**Fig. 9:**
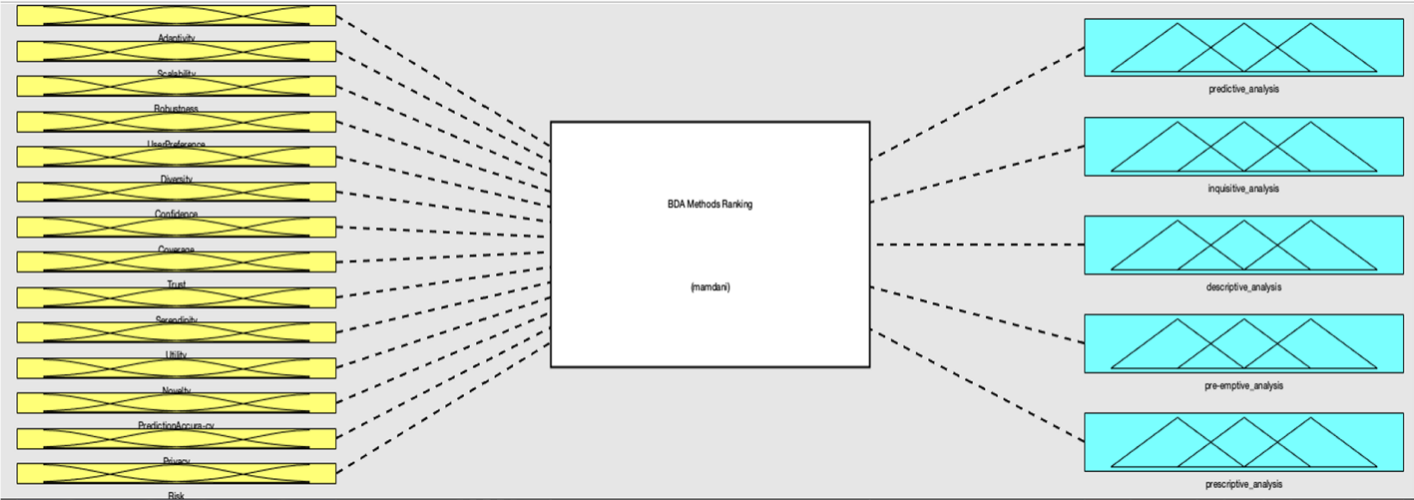
General view of BDA methods ranking’ FIS

**Fig. 10:**
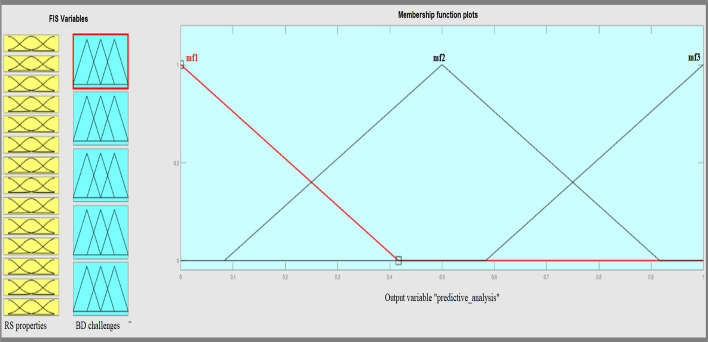
Fuzzy membership functions of input and output variables

This FIS ranks the BDA methods based on the RS properties. When an RS has the property of prediction accuracy then the predictive analysis is the best choice. For RSs with a high risk, the descriptive and preemptive analyses are good choices. The triangular fuzzy membership functions in three scales of the low, medium, and high are selected to deal with the fuzzy expression of both input and output variables (Fig. 9). The triangular membership function is the simplest MF for FISs with a high number of inputs and outputs and supports the consistency and comprehensiveness of rules (Fig. 10).

**Fig. 11:**
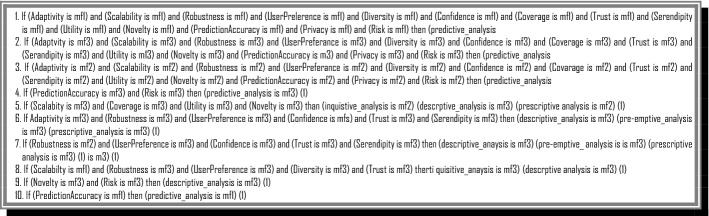
A part of rules in BDA methods ranking FIS

The rules of this FIS are designed based on the relation of RS properties and BDA methods. Figure 11 shows a part of the rules in this FIS.

## Results and discussion

We provide the results in four parts that are related to Q1, Q2, Q3, and statistical testing of results correspondingly. The results of fuzzy MCDM are used for designing rules in FISs so we did not use these results directly in a formula or method. According to the priority of RS properties, we focus on designing rules for the most important properties. Moreover, their related rules received higher weights in comparison with other properties.

### Fuzzy MCDM results—related to Q1

We used fuzzy AHP method for evaluating RS properties. This evaluation is based on our opinions rather than experiments. We study all RS properties and BD challenges in RSs before filling up the pairwise comparison matrix (Table 3).

**Table 3:** Pairwise comparing—14*14 matrix

RS properties	Adaptivity	Scalability	Robustness	User Preference	Diversity	Confidence	Coverage	Trust	Serendipity	Utility	Novelty	Prediction Accuracy	Privacy	Risk
Adaptivity	E	W	FS	VS	FW	VS	VS	W	VW	VS	VS	W	FW	FS
Scalability		E	VS	VS	S	S	VS	VS	S	VS	VS	VS	FS	VS
Robustness			E	E	W	W	E	W	VW	W	W	W	VW	W
User Preference				E	W	W	W	W	VW	E	W	VW	VW	E
Diversity					E	S	VS	S	W	S	S	S	E	S
Confidence						E	S	S	VW	S	W	W	VW	VS
Coverage							E	W	W	W	W	W	W	E
Trust								E	W	S	S	S	VW	S
Serendipity									E	VS	VS	VS	S	VS
Utility										E	W	W	VW	E
Novelty											E	W	VW	S
Prediction Accuracy												E	VW	S
Privacy													E	VS
Risk														E

Table 4 shows the fuzzified comparison matrix. We replaced the linguistic variables with their corresponding fuzzy numbers determined in Table 1.

**Table 4:** Fuzzy pairwise comparison matrix

RSs properties	Adaptivity	Scalability	Robustness	User Preference	Diversity	Confidence	Coverage	Trust	Serendipity	Utility	Novelty	Prediction Accuracy	Privacy	Risk
Adaptivity	(1, 1, 1)	(1, 1/3, 1/5)	(5, 7, 9)	(7, 9, 10)	(1/5, 1/7, 1/9)	(7, 9, 10)	(7, 9, 10)	(1, 1/3, 1/5)	(1/7, 1/9, 1/10)	(7, 9, 10)	(7, 9, 10)	(1, 1/3, 1/5)	(1/5, 1/7, 1/9)	(5, 7, 9)
Scalability		(1, 1, 1)	(7, 9, 10)	(7, 9, 10)	(1, 3, 5)	(1, 3, 5)	(7, 9, 10)	(7, 9, 10)	(1, 3, 5)	(7, 9, 10)	(7, 9, 10)	(7, 9, 10)	(5, 7, 9)	(7, 9, 10)
Robustness			(1, 1, 1)	(1, 1, 1)	(1, 1/3, 1/5)	(1, 1/3, 1/5)	(1, 1, 1)	(1, 1/3, 1/5)	(1/7, 1/9, 1/10)	(1, 1/3, 1/5)	(1, 1/3, 1/5)	(1, 1/3, 1/5)	(1/7, 1/9, 1/10)	(1, 1/3, 1/5)
User Preference				(1, 1, 1)	(1, 1/3, 1/5)	(1, 1/3, 1/5)	(1, 1/3, 1/5)	(1, 1/3, 1/5)	(1/7, 1/9, 1/10)	(1, 1, 1)	(1, 1/3, 1/5)	(1/7, 1/9, 1/10)	(1/7, 1/9, 1/10)	(1, 1, 1)
Diversity					(1, 1, 1)	(1, 3, 5)	(7, 9, 10)	(1, 3, 5)	(1, 1/3, 1/5)	(1, 3, 5)	(1, 3, 5)	(1, 3, 5)	(1, 1, 1)	(1, 3, 5)
Confidence						(1, 1, 1)	(1, 3, 5)	(1, 3, 5)	(1/7, 1/9, 1/10)	(1, 3, 5)	(1, 1/3, 1/5)	(1, 1/3, 1/5)	(1/7, 1/9, 1/10)	(7, 9, 10)
Coverage							(1, 1, 1)	(1, 1/3, 1/5)	(1, 1/3, 1/5)	(1, 1/3, 1/5)	(1, 1/3, 1/5)	(1, 1/3, 1/5)	(1, 1/3, 1/5)	(1, 1, 1)
Trust								(1, 1, 1)	(1, 1/3, 1/5)	(1, 3, 5)	(1, 3, 5)	(1, 3, 5)	(1/7, 1/9, 1/10)	(1, 3, 5)
Serendipity									(1, 1, 1)	(7, 9, 10)	(7, 9, 10)	(7, 9, 10)	(1, 3, 5)	(7, 9, 10)
Utility										(1, 1, 1)	(1, 1/3, 1/5)	(1, 1/3, 1/5)	(1/7, 1/9, 1/10)	(1, 1, 1)
Novelty											(1, 1, 1)	(1, 1/3, 1/5)	(1/7, 1/9, 1/10)	(1, 3, 5)
Prediction Accuracy												(1, 1, 1)	(1/7, 1/9, 1/10)	(1, 3, 5)
Privacy													(1, 1, 1)	(7, 9, 10)
Risk														(1, 1, 1)

The fuzzy numbers are defuzzified through centroid defuzzification method and presented in Table 5.

**Table 5:** Defuzzified pairwise comparison matrix

1	1/3	7	9	1/7	9	9	1/3	1/9	9	9	1/3	1/7	7
	1	9	9	3	3	9	9	3	9	9	9	7	9
		1	1	1/3	1/3	1	1/3	1/9	1/3	1/3	1/3	1/9	1/3
			1	1/3	1/3	1/3	1/3	1/9	1	1/3	1/9	1/9	1
				1	3	9	3	1/3	3	3	3	1	3
					1	3	3	1/9	3	1/3	1/3	1/9	9
						1	1/3	3	1/3	1/3	1/3	1/3	1
							1	3	3	3	3	1/9	3
								1	9	9	9	3	9
									1	1/3	1/3	1/9	1
										1	1/3	1/9	3
											1	1/9	3
												1	9
													1

We obtained the eigenvector of the defuzzified pairwise comparison matrix. It is considered the RS properties important in Table 6.

**Table 6:** RS properties ranking

RS properties Modes	Importance degree
Adaptivity	0.60
Scalability	0.98
Robustness	0.10
User Preference	0.27
Diversity	0.72
Confidence	0.53
Coverage	0.06
Trust	0.76
Serendipity	0.94
Utility	0.34
Novelty	0.45
Prediction Accuracy	0.71
Privacy	0.94
Risk	0.76

### BD challenges ranking—related to Q2

**Fig. 12:**
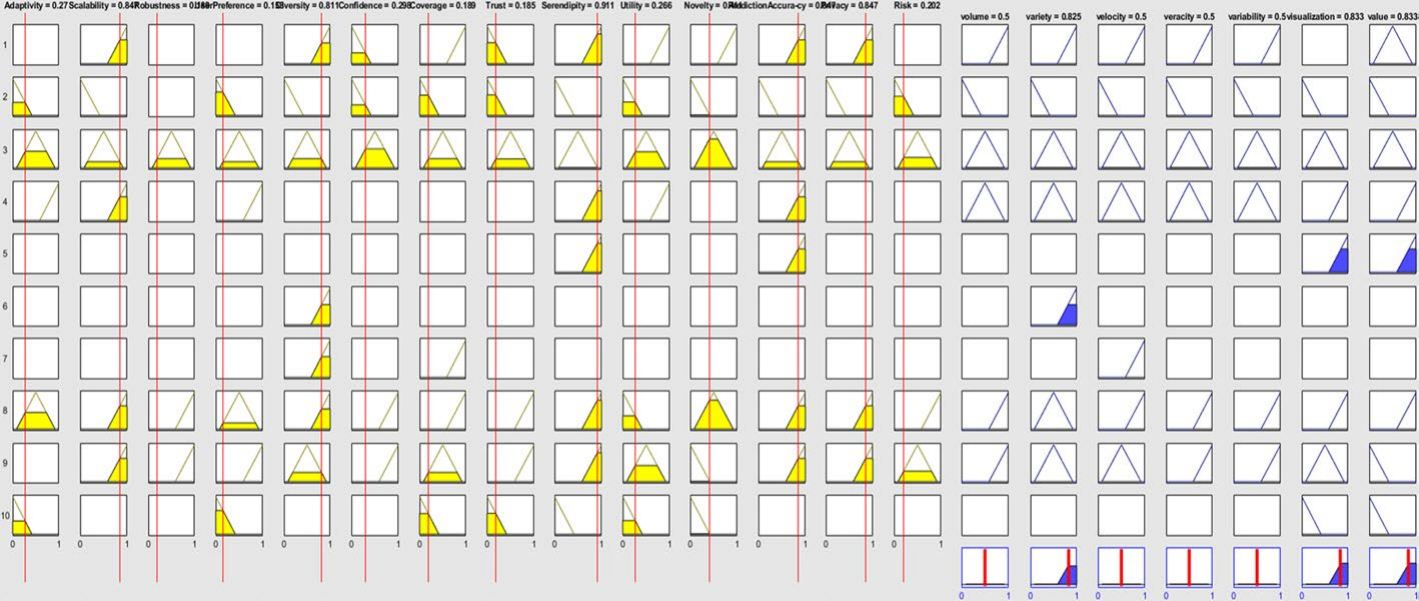
Ranking of BD challenges for a specific collaborative system

A collaborative RS with the presented degree of properties is the input of FIS system (Fig. 12). The output shows the medium severity for volume, variety, velocity, variability challenges (0.5) and the high degree of severity for visualization and value challenges (0.83).

Input: [0.2705;0.8468;0.1885;0.1532;0.8115;0.2984;0.1885;0.1855;0.9113;0.2661;0.4113;0.8468;0.8468;0.2016].

**Fig. 13:**
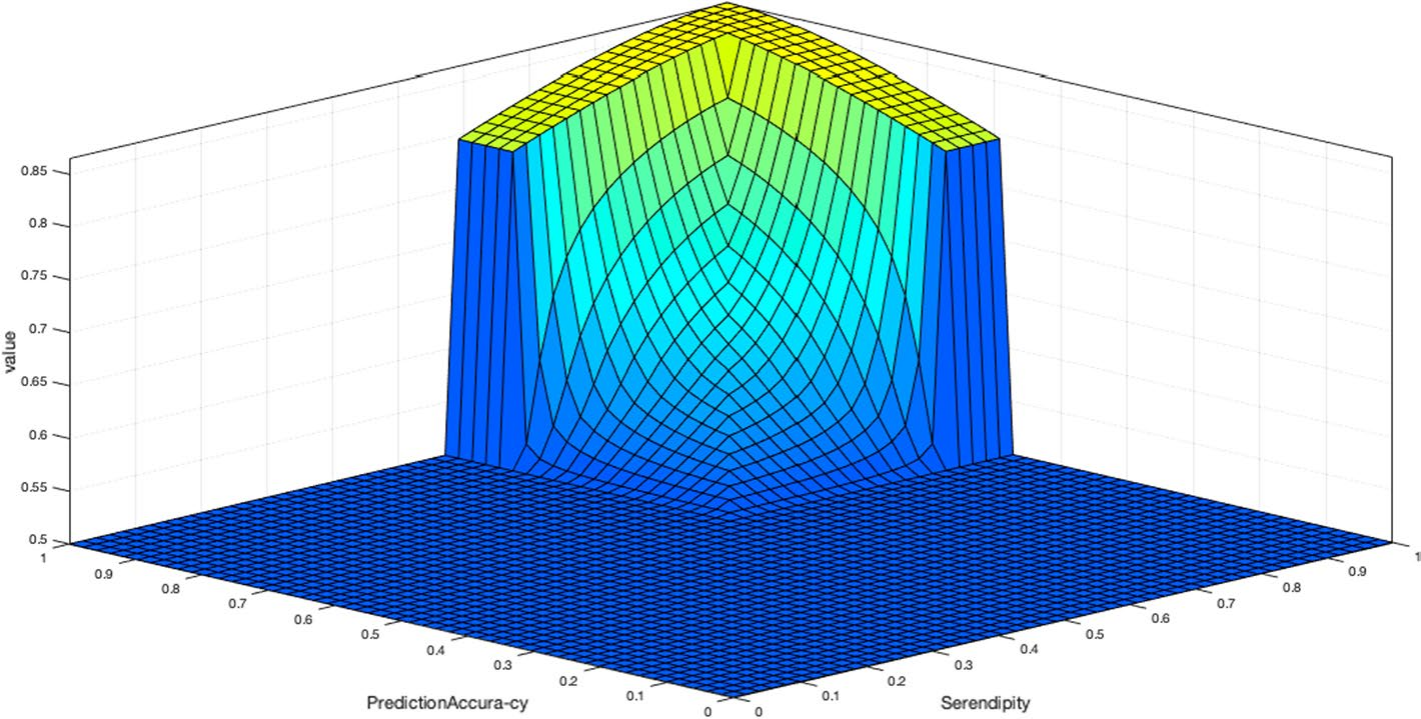
The relation of two RS properties and the degree of severity in value (BD challenge)

The results of pairwise relation between every two inputs show that how every two inputs affect outputs. Figure 13 shows that the prediction accuracy and serendipity have the positive effect on value challenge. Therefore, we conclude that when there is a RS with the prediction accuracy requirement and serendipity of users then the most important challenge that we need to consider is value challenge.

### BDA methods ranking (Q3)

**Fig. 14:**
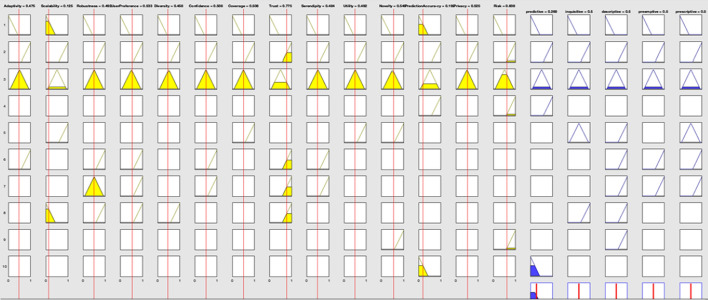
The view of rules for a group RS

Group RSs provide recommendations for a group instead of an individual person and they have low scalability and privacy. The BDA ranking is the most important result that can recommend the best BDA method to scientists for analyzing BD in RSs. Figure 14 presents the ranking of BDA methods for a group RS. In this RS, the scalability and prediction accuracy are low, and trust is high, so the predictive analysis got the low degree (0.3) and the rest of the method got a moderate degree (0.5).

**Fig. 15:**
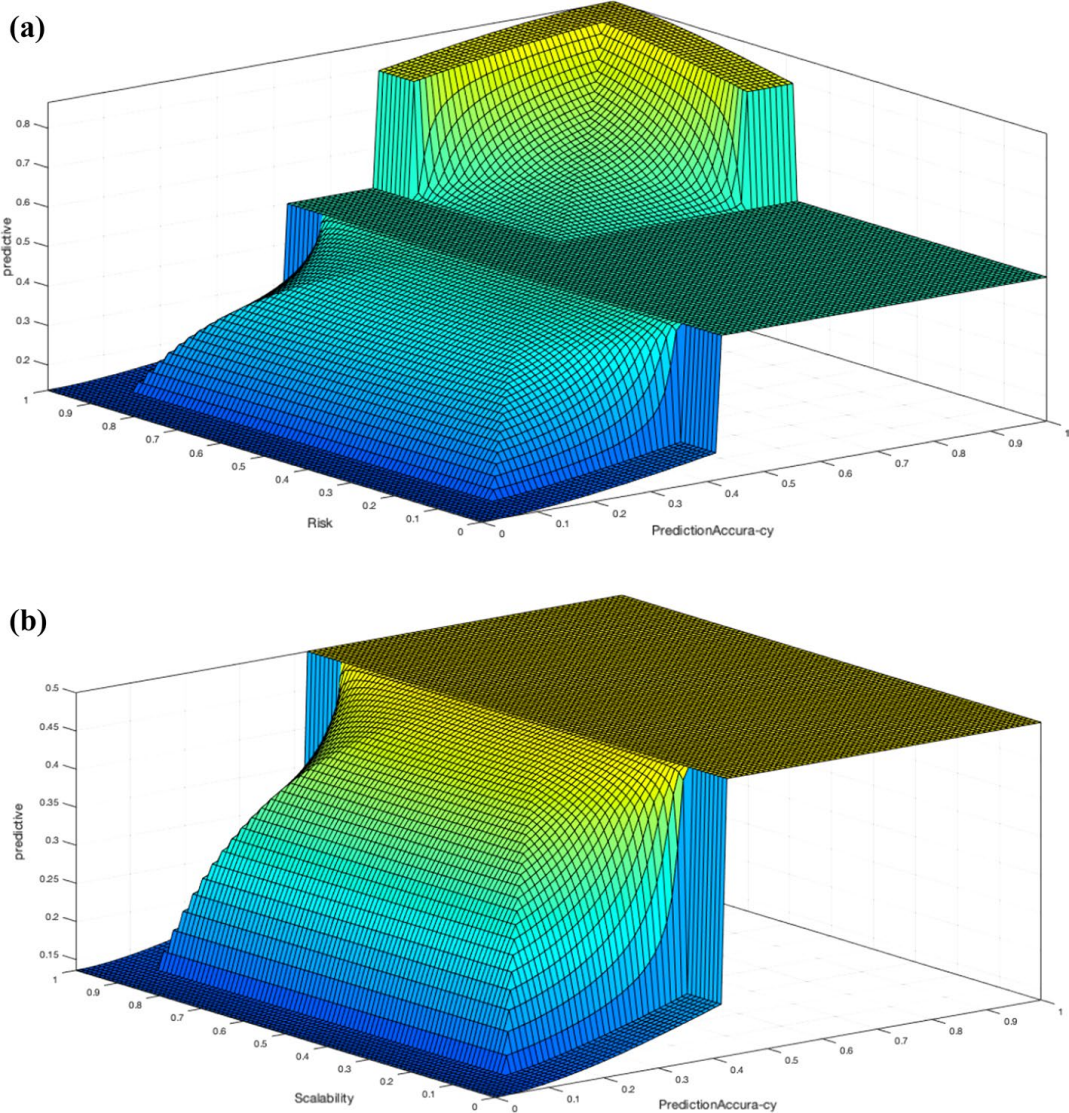
**a** The relation of two RS properties and the degree of severity in predictive analysis (BDA method). **b** The relation of two RS properties and the degree of severity in predictive analysis (BDA method)

Figure 15a and b shows that there is positive relation between risk and predict accuracy with predictive analysis. Also, there is a positive relation between scalability and predict accuracy with predictive analysis. Therefore, a RS with high risk and the requirement of predict accuracy needs to be analyzed through a predictive analysis.

### Statistical analysis

We apply statistical analysis to prove the significant relation between FIS results and known real world cases.

The used variables are:

1.RS properties (independent, ratio)

2.BD challenges (dependent, ratio)

3.BDA methods (dependent, ratio)

In SPSS software, the Pearson correlation coefficient (CC) analyzes the relationship between rankings generated by FISs and real-world cases. This coefficient is a statistical tool to determine the type and extent of the relationship between variables and shows the correlation between two variables. Here, this method is used to determine the correlation between two variables. The correlation coefficient (r) shows how the data of a scatter are placed in a straight line.

The results of CC with p < 0.5 and r > 0.8 for both FISs show the significant relationship between FISs results and real-world cases. Therefore, the proposed model can efficiently be applied for ranking BD challenges and BDA methods.

## Conclusion

The evaluation of BD challenges and BDA methods is a decision-making issue, and it has a strong influence on the overall improvement of BD RSs. Automation of ranking BD challenges and BDA methods helps data scientists and researchers to select the most proper techniques to deal with any BD RS. The data analysis of BA is a costly process, and the developers need to predict the property of a method before the adoption of that method. The BD challenges and BDA methods’evaluation include qualitative criteria which are RS properties. Moreover, the importance of RS properties in the ranking of challenges and methods is important. Therefore, an efficient and dynamic evaluation method is necessary to deal with BD in RS. In this study, we proposed an integrated model with three phases. The fuzzy method is integrated with MCDM methods to increase the accuracy of evaluation. The first phase was an investigation of BD and RS ontology. In this phase, we determined the BD challenges and RS properties based on a literature review. The second phase was the weighting of RS properties. In the third phase, we implemented two FISs for automation of ranking of challenges and methods for any specific RS. To the best of our knowledge, an automotive BD challenge and method evaluation is a novel system in BD RSs. The results show that the proposed model has an accurate prediction of challenges and ranking methods for RS such as collaborative RS. The proposed model can apply to all types of BD RSs. Future studies may improve the model with FIS for the evaluation of BD tools.

## Data Availability

Available if needed.
